# Structural and functional studies of *Porphyromonas gingivalis* fimbrial proteins

**DOI:** 10.1080/20002297.2017.1325209

**Published:** 2017-06-01

**Authors:** Karina Persson, Michael Hall

**Affiliations:** ^a^ Department of Chemistry, Umeå University, Umeå, SE-901 87, Sweden

## Abstract

*Porphyromonas gingivalis* expresses two forms of fimbriae, FimA and Mfa1. Each fimbria consists of five proteins; FimA-E and Mfa1-5. While the assembly of the type-1 fimbriae from *Escherichia coli* is well studied; the chaperone-usher pathway, very little is known about the polymerization of *P. gingivalis* fimbriae. The *P. gingivalis* fimbrial proteins form lipidated precursors that have an N-terminal extension, which is not found in the mature protein.

In order to obtain an understanding of the structure, function and assembly mechanism of the *P. gingivalis* fimbriae we are studying the Mfa proteins using X-ray crystallography. The Mfa proteins have been crystallized in their precursor forms and their crystal structures reveal that the proteins are structurally related despite low sequence similarity. The proteins consist of two β-sandwich domains where the N-terminal extension forms a β-strand that is tightly integrated in the first β-sheet. An arginine, recognized by a protease, is exposed on a long flexible loop.

The structures raise questions if the N-terminal extension functions as an internal chaperone that stabilizes the protein before polymerization is initiated. We further speculate which structural part of the protein that can function as a donor strand upon fimbrial polymerization.

